# Persistent infections after natural transmission of bovine viral diarrhoea virus from cattle to goats and among goats

**DOI:** 10.1186/1297-9716-44-32

**Published:** 2013-05-15

**Authors:** Claudia Bachofen, Hans-Rudolf Vogt, Hanspeter Stalder, Tanja Mathys, Reto Zanoni, Monika Hilbe, Matthias Schweizer, Ernst Peterhans

**Affiliations:** 1 Institute of Veterinary Virology, University of Bern, Länggassstr. 122, P.O. Box 8466, CH-3001 Bern, Switzerland; 2 Institute of Veterinary Pathology, University of Zürich, Winterthurerstr. 268, CH-8057 Zürich, Switzerland

## Abstract

Bovine viral diarrhoea virus (BVDV) is an economically important pathogen of cattle worldwide. Infection of a pregnant animal may lead to persistent infection of the foetus and birth of a persistently infected (PI) calf that sheds the virus throughout its life. However, BVD viruses are not strictly species specific. BVDV has been isolated from many domesticated and wild ruminants. This is of practical importance as virus reservoirs in non-bovine hosts may hamper BVDV control in cattle. A goat given as a social companion to a BVDV PI calf gave birth to a PI goat kid. In order to test if goat to goat infections were possible, seronegative pregnant goats were exposed to the PI goat. In parallel, seronegative pregnant goats were kept together with the PI calf. Only the goat to goat transmission resulted in the birth of a next generation of BVDV PI kids whereas all goats kept together with the PI calf aborted. To our knowledge, this is the first report which shows that a PI goat cannot only transmit BVD virus to other goats but that such transmission may indeed lead to the birth of a second generation of PI goats. Genetic analyses indicated that establishment in the new host species may be associated with step-wise adaptations in the viral genome. Thus, goats have the potential to be a reservoir for BVDV. However, the PI goats showed growth retardation and anaemia and their survival under natural conditions remains questionable.

## Introduction

Bovine viral diarrhoea (BVD) virus is one of the economically most important cattle pathogens world-wide. Together with border disease virus (BDV) of sheep and classical swine fever virus (CSFV) of pigs it forms the genus *Pestivirus* of the family *Flaviviridae*[1]. The success of BVDV is due to its ability to cause two types of infection. Pregnant animals acutely infected between the second and fourth month of gestation may generate persistently infected (PI) offspring. Such PI animals are immunotolerant specifically to the infecting virus strain [2]. They produce neither anti-BVDV antibodies nor BVD virus-specific T-cell responses; they do, however, spread the virus for life via saliva and other secretions and are the most important source of infection for other animals [3]. Therefore, programmes to eradicate BVDV are primarily based on detection and removal of PI animals [4]. However, the focus on bovines alone in BVD eradication programmes may be problematic because BVD virus is known to also infect other wild and domestic species of the artiodactyla, as shown by serological studies [5-7]. Besides cattle, evidence for persistent infection has been proven in at least seven species (sheep, pigs, alpaca, white-tailed deer, eland, mouse deer, and American mountain goat) [8-14].

Among these species, domestic small ruminants are of main interest as potential virus reservoir. Sheep PI with BVDV have been reported frequently, indicating that the virus is easily transmitted from cattle to sheep [8,15-21]. By contrast, transmission of BVDV to goats is less clear. Prevalence of pestivirus antibodies in goats is reported to range from of 2–25%, with the majority of reports being between 10–16% [22-26]. Herd seroprevalences are highly variable. In Austria, the average flock prevalence was reported to be 31.3% [24] but may be as high as 83% [27]. In several studies, the seroprevalence in goats was found to be significantly higher in herds that had contact to cattle [22,24,27]. Interestingly, compared to sheep where BDV antibodies are most prevalent, pestivirus antibodies in goats are more often not clearly specifiable or are rather directed against BVDV [25,27].

Thus, observations from experimental or confirmed natural infections may be more informative for assessing the role and effects of BVDV infections in goats. In the majority of cases, infections of pregnant goats with BDV, BVDV-1 and BVDV-2 seem to result in abortions and stillbirths with an overall reproductive failure of up to 82% [28-31]. However, viable virus-positive offspring has also been reported, albeit rarely. Løken and Bjerkas [28] detected three virus-positive kids after experimental infection with BVDV of 21 pregnant goats. Virus was also isolated from 4 kids after 276 female goats were accidentally infected by BVDV via a contaminated Orf-vaccine [29]. BVDV-2 has been isolated from one goat in India [32] and one goat in South Korea [33]. A serological and virological survey in small ruminants in Austria revealed one BVDV-1 positive goat [34]. However, due to a lack of repeated testing, in some cases it remained unclear if the infection was indeed persistent. Clinical signs of persistent infection with BVDV in goats may be quite diverse. In some cases goats remained apparently healthy but, more often, kids were weak and showed border disease-like symptoms and ill thrift [28,29,35]. Infection of neonatal kids with both BDV and BVDV resulted in growth retardation and histological changes in the CNS [36]. Due to the low prevalence of PI goats and their poor survival rate, goats are not expected to play an important role in the epidemiology of BVDV [37].

In this work, we report on a PI goat kid born to a goat kept together with a BVDV PI calf. In order to analyse the potential of BVDV to establish a chain of infection within the goat population, we exposed seronegative pregnant goats to this initial PI goat. In addition, we repeated the cattle to goat transmission. However, only the goat to goat transmission resulted in the birth of a next generation of BVDV PI kids. Hence, a second round of persistent infection was initiated in a species which is generally considered to be a “dead end” for the spread of BVDV. Our results indicate that BVDV may have the potential to persist in the goat population even in the absence of contact to PI cattle and that establishment in the new host species may be associated with step-wise adaptations in the viral genome.

## Materials and methods

### Generation of the initial PI goat

A female adult goat of the Saanen breed was housed as a social companion with a calf PI with BVDV. The PI animal was a female calf of the Swiss Rotfleck breed and three weeks of age when joined by the goat. Four months after the first exposure to the PI calf a small but healthy male goat kit was born. Therefore, infection of the dam must have occurred after day 37 of gestation. Blood samples were taken from the goat kid within 1–2 h after birth and 2 days later and tested for the presence of viral RNA by RT-PCR.

### Experimentally exposed goats

All goats used for the experimental exposures were healthy adult females of the Saanen breed originating from a flock of experimental animals tested free of small ruminant lentiviruses and regularly treated against ecto- and endoparasites. They had been repeatedly tested negative of pestiviruses and antibodies to pestiviruses as analyzed by a pan-pesti RT-PCR and by antibody capture ELISA, respectively, as described below.

### Set up for in vivo transmission experiments

Group A: Five goats (A1-A5, Table 1 and Additional file 1) were exposed for seven days to the PI calf that was the source of infection for the initial PI goat. The calf was now a heifer of 22 months. The animals were housed together in an isolation barn of about 15m^2^ at the University of Bern. Due to the relatively limited space available in the isolation barn alongside the heifer, goats were successively exposed pair-wise and for only seven days. The fifth animal was commingled thereafter with the PI animal alone for seven days. Blood samples (EDTA and non-anticoagulated) were taken daily for the first 14 days and later weekly until day 42. The clinical status of the animals and the body temperature was measured daily for the first 14 days. Gestation was confirmed by ultrasound on days 36 and 55 after mating. The goats A1, 2, 3 and 4 were on days 38–45 of gestation (which represents the time point we assume that the initial PI goat was generated) when they were exposed to the PI heifer. The fifth animal (A5) was on days 59–66 of gestation during exposure (Table 1).

**Table 1:** Results of experimental exposure of pregnant, seronegative goats to BVDV

	**Exposure**			**Viremia**		**Fever**		**Seroconversion**		**Possible sign of abortion**		**BVD status of kids/ foetuses**	**Remarks**
**Goat**	**to:**	**Duration (d)**	**d.o.g.**	**d.a.f.e.**	**d.o.g.**	**d.a.f.e.**	**d.o.g.**	**d.a.f.e.**	**d.o.g.**	**d.a.f.e.**	**d.o.g.**		
A1	PI heifer	7	38–45	-	-	-	-	21	59	28	66	n.a.	Aborted foetus not detected
A2	PI heifer	7	38–45	-	-	11, 12	49, 50	21	59	111	149	n.a.	Aborted foetus not detected
A3	PI heifer	7	38–45	11	49	-	-	28	66	108	146	+ (1)	Aborted foetus (A3_1) BVDV positive
A4	PI heifer	7	38–45	7, 9	45, 47	8	46	21	59	104	142	n.a.	Aborted foetus not detected
A5	PI heifer	7	59–66	-	-	-	-	21	80	-	-	n.a.	Aborted foetus not detected
B1	PI goat	21	42–63	-	-	n.d.	n.d.	28	70	(105)*	(147)*	+ (3)	Three dead BVDV positive kids (B1_1, 2, 3)
B2	PI goat	21	17–38	-	-	n.d.	n.d.	42	59	-	-	+ (2)	Two living BVDV PI kids (B2_1, 2)
B3	PI goat	21	38–59	-	-	n.d.	n.d.	-	-	-	-	-	One healthy, BVDV negative kid

d.o.g. = days of gestation; d.a.f.e. = days after first exposure; n.d. = not done; n.a. = not applicable; *goat died shortly before parturition.

Group B: Three goats (B1-B3) were exposed to the PI goat that was 18 months old at the beginning of exposure. The three goats were housed together with the PI goat for 21 days in a stable some 10 km apart of group A. The barn was of about the same size as that of group A but due to the longer exposure time and for animal welfare reasons, access to an enclosed open-air paddock was allowed. After the exposure time the PI goat was removed from the group. Blood samples were taken on a weekly basis until day 42. The goats of group B were at days 42–63 (B1), 17–38 (B2) and 38–59 (B3) of gestation when they were exposed to the PI goat.

A graphical overview of the animal groups and individual animal numbers can be found in the Additional file 1. All animals were kept under traditional, agricultural conditions and in compliance with the Swiss Animal Protection Act (1978). All samplings and treatments were performed according to the principles of good veterinary practice which were considered non-regulated procedures by the ethics committee of the canton of Bern.

### Real time-RT-PCR

For RNA isolation from EDTA blood the QIAamp RNA Blood Mini Kit and for isolation from tissue samples the RNeasy Mini Kit were used (both from Qiagen, Hombrechtikon, Switzerland) according to the manufacturer’s instruction.

The real-time RT-PCR was applied as previously described, using primers and probes located in the 5’ untranslated region (5'utr) of the viral genome [38]. Briefly, the TaqMan One-Step RT-PCR Master Mix Kit (Applied Biosystems, Life Technologies Europe BV, Zug, Switzerland) was used, the primers and probes as well as 5 μL of the isolated RNA added in a final volume of 25 μL and the reaction run in a ABI Prism 7700 Sequence Detection System using the following programme: 30 min 48°C (RT-step), 10 min 95°C (activating AmpliTaq Gold), and 45 cycles of 15 s 95°C and 1 min 60°C.

### Direct sequencing

We used serum samples from the PI heifer and the initial PI goat taken two weeks prior to the beginning of the exposure experiment, and abdominal fluid from the aborted foetus (A3_1) for comparison of the deduced amino acid sequence of the viral envelope protein E2. Viral RNA from spleen samples of the stillborn goat kids (B1_1, 2, 3) and from uterus tissue samples of the dam (B1) was also sequenced. From the two living PI kids we used pre-colostral blood samples. In addition, in vitro passaged virus from the PI heifer and the PI goat was sequenced. Both sera had been passaged ten times in homologous and heterologous cells (bovine turbinate and goat synovial membrane cells) in parallel as described [39].

RNA isolation and conventional RT-PCR followed by sequencing of the viral genome in the 5’utr and the envelope protein E2 coding region was performed as previously described [40]. Briefly, QIAGEN spin columns were used for the RNA isolations followed by one-step RT-PCR reactions using the One Step RT-PCR kit from QIAGEN. For the 5’utr sequencing we used the pan-pesti primer pair 324/326 [41]. For the highly variable E2 coding region we designed the primer pair 232f/234r based on published full length sequences of BVDV 1 viruses. Their position in the BVDV NADL type strain is as follows: 232f (5’-GTYTAAGKCCYYARTGGTGGC-3’) 2244–2265, 234r (5’-RVTCRTCRCTRAGRAYDAGGTA-3’), 3671–3692. For sequence analysis and comparison, the SeqMan software from the Laser gene suite (DNASTAR Inc., Madison WI, USA) as well as the Clone Manager software (Scientific & Educational software, Cary NC, USA) were used.

The virus of the PI heifer has been described previously as strain “CH-Maria” [40] and has the Gen Bank accession numbers EU180028 (5’utr) and EU180048 (E2).

### Antibody capture ELISA

A biphasic in-house ELISA was used as previously described [42] to detect antibodies against the conserved NS3 protein of pestiviruses in the goat sera. Briefly, ELISA microtitre plates (Maxisorp, A/S Nunc, Kamstrub, Denmark) were coated with antigen derived from bovine turbinate cells. For this purpose, cell cultures that were either infected with the cytopathic BVDV strain R1935/72 (Oregon C24V, subgenotype BVDV-1a) or that remained non-infected were freeze-thawed three times, centrifuged and the pellet re-suspended and incubated in phosphate buffered saline (PBS). In order to obtain mainly non-structural viral proteins, 2% Tween 20 was added. Columns were coated alternatively with antigen derived from infected and non-infected cell cultures to control for unspecific binding. Sera were diluted 1:10 in blocking buffer (PBS, 0.05% Tween 20 and 1% milk powder) before being added to the coat. As a conjugate, Protein-G-Peroxidase (Bioreba AG, Basel, Switzerland) was used for non-bovine species. To visualize bound antibodies, the substrate ABTS (2,2’-azino-di-(3-ethyl benzthiazoline-6- sulphonic acid); Roche Diagnostics, Rotkreuz, Switzerland) was added and the intensity of the staining was measured by an ELISA reader at 405nm.

### Immunohistochemistry

Immunohistological analyses for BVDV antigen detection were performed as previously described [38]. A snap frozen skin biopsy and paraffin embedded bone section (femur) were analysed using the C16 and 15c5 pan-pesti antibodies, respectively.

## Results

**Figure 1:**
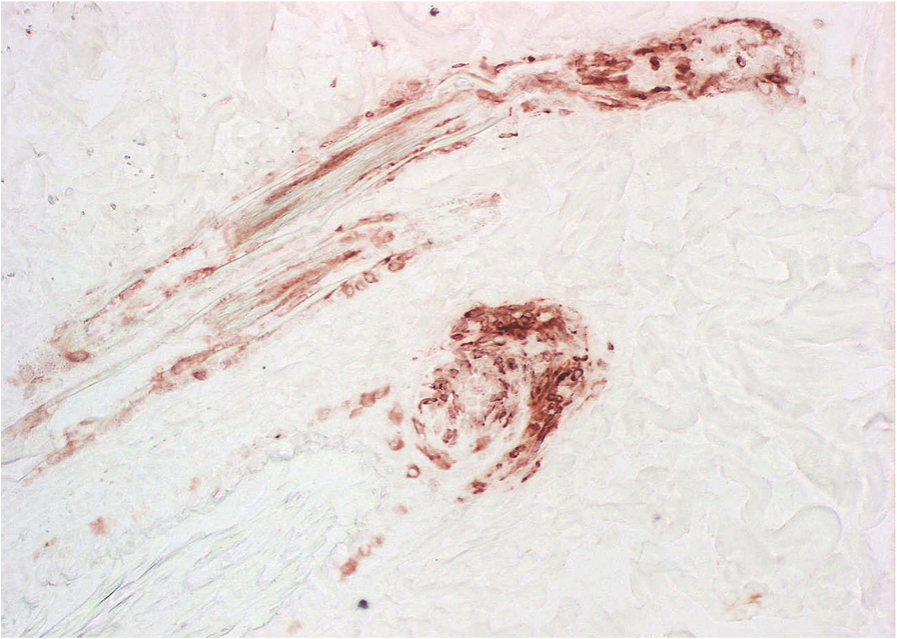
**Immunohistochemistry of a skin biopsy of the initial PI goat.** The immunohistological analysis of a snap frozen skin biopsy of the initial PI goat shows the presence of BVDV antigen (red staining) in epithelial cells of the hair root shaft as typically seen in PI cattle [48]. C16 antibody, 20× magnified.

A goat being housed as a social companion with a PI calf delivered a small but healthy male kid, thereafter referred to as initial PI goat. Blood samples taken from the kid were positive for viral RNA. Immunohistochemistry of a skin biopsy showed the antigen distribution typical for PI animals [43] (Figure 1). Virus was isolated from serum, saliva, nasal secretion, tears and hair of the goat kid (data not shown). The virus titre in serum was 5.6 × 10^4^ TCID_50_/mL. A serum sample taken from the PI heifer at the same time point showed a titre of 2.1 × 10^5^ TCID_50_/mL. Sequencing revealed the viruses to be of the BVDV 1e subgroup and the sequences of the 5’utr fragment were identical (data not shown).

### Goats exposed to the PI heifer (group A)

The clinical, serological and virological findings of the pregnant goats exposed to the PI heifer are summarised in Table 1. The health status of the five goats was analysed daily during the first 14 days. Animal A4 had a temperature of 39.9°C on day 8, goat A2 40.2°C on day 11 and 40.4°C on day 12. No loss of appetite or clinical signs such as diarrhoea, nasal discharge or coughing was observed. EDTA blood was tested by real-time RT-PCR to detect viremia. Two animals showed weak positive results with CT values above 35; one on day 11 (A3) and the other (A4) on days seven and nine after first exposure. The latter was the same animal that also had a fever at day eight. Serum samples analysed by antibody capture ELISA showed that four out of five animals of group A seroconverted between days 14 and 21 after initial exposure. All goats were seropositive by day 28 after first exposure, which indicated that they had been infected by the PI heifer. This had serious consequences for gestation: All animals aborted, most of them apparently in late gestation. All animals were still in gestation when being checked by ultrasound on day 55 of gestation, ten days after termination of the exposure. On day 66 of gestation, bloody vaginal secretion was observed from goat A1 and it returned to oestrus 12 days later. The other four goats remained clinically inconspicuous. However, no udder formation was observed by day 120 of gestation. In three cases bloody vaginal secretion was observed on days 146 (A3), 149 (A2) and 142 (A4) of gestation (with the normal duration of gestation in goats being on average 150 days). Only in one case the aborted foetus which showed signs of mummification was available for analysis (A3_1). It was detected on day 146 of gestation but the condition, size and development of the foetus indicated that it had died as early as around day 90 of gestation. We were able to detect BVD viral RNA in abdominal fluid and several organs of this foetus by RT-PCR. Bacteriological analyses for Brucella, Chlamydia, Coxiella and Neospora were negative. Sequencing in the BVDV 5’utr proved the virus to be identical to that of the PI calf and goat. No observations were made that pointed to the date of abortion in animal A5.

### Goats exposed to the PI goat (group B)

No viremia was detected in the three goats exposed to the initial PI goat (Table 1). This is not unexpected as blood samples were taken only once a week and viremia during acute infection with BVDV is usually short-lived [44]. The results of the antibody capture ELISA revealed that two goats had seroconverted; one between days 21 and 28 (B1), the other between days 35 and 42 after first exposure (B2). One goat (B3) remained antibody negative and gave birth to a healthy BVDV-free kid. Unfortunately, the goat B1 died shortly before the expected date of parturition. The post-mortem examination revealed a severe intestinal volvulus with haemorrhagic infarction as the most likely cause. It had carried three kids that however did not survive. They were well developed and RT-PCR showed that all of them were strongly BVDV positive in serum and all organs analysed. No antibodies against BVDV were detected. The goat B2 gave birth to two female kids (B2_1 and B2_2). Pre-colostral blood samples were taken and both were antibody negative but strongly BVDV positive by RT-PCR. Blood samples taken over the next few weeks were tested by conventional and real-time RT-PCR and were always positive.

### Development of PI goats

The initial PI goat was undersized but stayed healthy until the age of 19 months when it developed severe untreatable anaemia and had to be euthanized at the age of 22 months.

**Figure 2:**
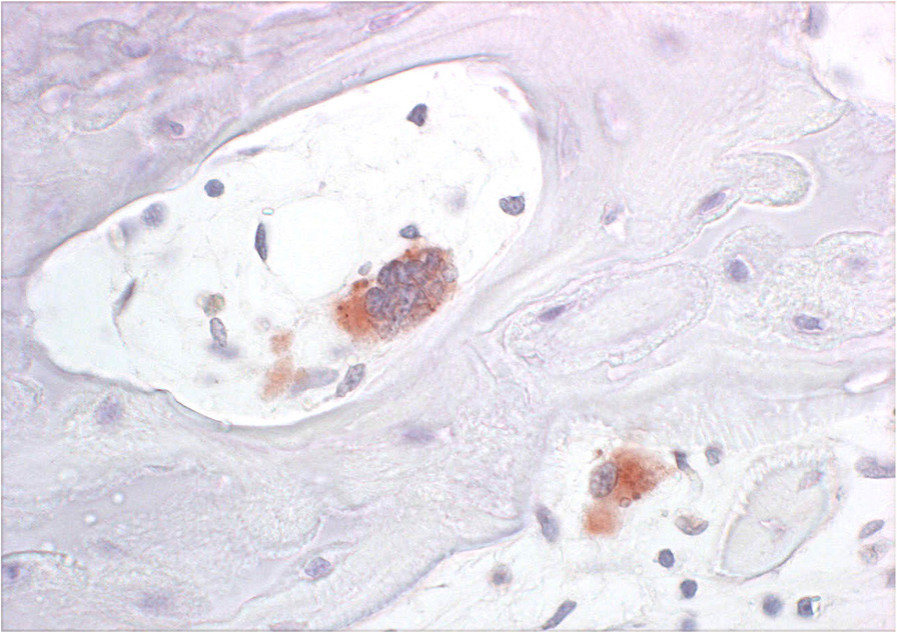
**Immunohistochemistry of the femur of PI goat B2_2.** The immunohistological analysis of a paraffin-embedded femur section of the PI goat B2_2 shows the presence of BVDV antigen (red staining) in the cytoplasm of an osteoblast (top) and an osteoclast (bottom). 15c5 antibody, 40× magnified.

B2_1 and 2 were also undersized and showed slight tremor and ataxia during the first few days of life. A video sequence showing the two PI kids at 3 hours and 3 weeks of age is given as Additional file 2. They recovered and developed quite well in the following weeks, although they remained undersized. Their health started to deteriorate at the age of around 7 weeks when they developed severe anaemia with haematocrit (HCT) values dropping below 10%. Transfusion of blood from their mother improved the situation only temporarily. Clinical, parasitological and haematological analyses did not reveal a clear cause for the anaemia. A bone marrow biopsy analysed at the Institute for Clinical Diagnostics of the University of Bern revealed severe acute hypoplastic anaemia with gelatinous bone marrow and virtually no erythropoiesis. All other organ functions did not seem to be impaired. Whereas B2_1 had to be euthanized due to the anaemia at 10 weeks of age, B2_2 recovered clinically but the HCT remained low at 13–15%. However, the animal had to be euthanized at the age of 15 months. Histological bone sections from the femur showed normal bone trabeculae and growth plates but the bone marrow showed reduced erythropoiesis and general atrophy with mild fibrosis. Multifocal myelopoiesis was present. Due to the reduction of erythropoiesis the ratio to the myelopoiesis was enhanced in favour of the latter. Osteocytes, osteoblasts and osteoclasts (Figure 2), chondrocytes, endothelial cells and single megakaryocytes stained positive for BVDV antigen.

### Analysis of viral genomic sequences

**Figure 3:**
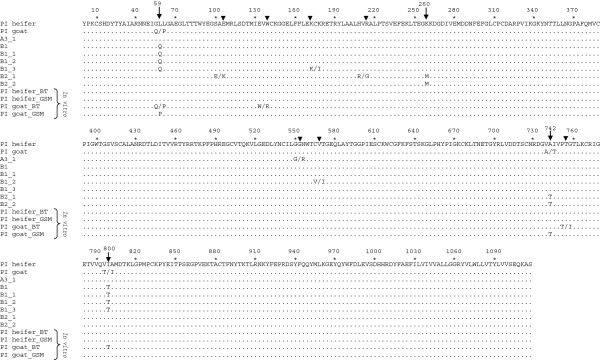
**Deduced amino acid alignment of the BVDV E2 coding region.** The coding region for the envelope glycoprotein E2 was determined (see Additional file 3) and the deduced amino acid sequences of viruses originating from cattle to goat and goat to goat infections were compared to the virus of the PI heifer. In addition, E2 amino acid sequences from viruses from the PI heifer and the initial PI goat (termed “PI goat”) that had been passaged in vitro in bovine turbinate (BT) and goat synovial membrane cells (GSM) are included. Dots represent identical amino acids. Positions of amino acid changes are highlighted by arrows. Numbering refers to the nucleotide positions as depicted in Additional file 3.

All sequences analysed were identical in the 246 nt long fragment of the conserved 5’utr (data not shown). By contrast, differences were apparent in the more variable coding region for the viral envelope glycoprotein E2 (Additional file 3). We compared the deduced amino acid sequence of the viral E2 protein from all PI animals that originated from cattle to goat transmissions and from the goat to goat transmission (Figure 3). Two samples originated from cattle to goat infections (i.e., serum from the initial PI goat and abdominal fluid from the aborted foetus A3_1) and six from goat to goat transmissions (uterus from goat B1, spleen from the dead triplet kids B1_1, 2, 3 and serum from the kids B2_1 and 2). In most sequences, single nucleotide ambiguities were present that could potentially lead to amino acid changes (Figure 3, triangles). However, only at four positions the same changes were observed in several viruses (Figure 3, arrows). Changes in nucleotide 59 led to a switch from a leucine (L) in the PI heifer to an ambiguity between glutamine (Q) and proline (P) in the initial PI goat and finally to a Q in the three stillborn kids and their mother. A change at nucleotide position 260 led to a switch from lysine (K) (in all other sequences) to methionine (M) in the two living PI animals (B2_1 and B2_2). At nucleotide 742, an ambiguity between alanine (A) (present in the PI heifer) and threonine (T) was observed in the initial PI goat. In the two living PI kids (infected by the initial PI goat) only the T was present. Finally, at nucleotide 800, the virus of the initial PI goat had an ambiguity between isoleucine (I) (present in the PI heifer) and T whereas in the stillborn kids and their mother it was clearly a T.

Interestingly, three out of these four positions also showed up in sequences from an in vitro passaging experiment performed with serum from the PI heifer and the PI goat [39]. After 10 passages in bovine turbinate (BT) or goat synovial membrane (GSM) cells, the virus from the initial PI goat revealed amino acid changes at positions 59 (in BT and GSM cells), 742 (GSM cells only) and 800 (BT cells only) as observed after goat to goat transmissions. By contrast, the virus from the PI heifer showed no amino acid changes after in vitro passaging in homologous (BT) and heterologous (GSM) cells (Figure 3).

## Discussion

BVDV is known to cross the species barrier with relative ease and the occurrence of BVDV positive offspring in small ruminants has been reported before [15,16,20,32-34]. However, in order to estimate the role of these animals as a potential reservoir for BVDV it is essential to know if the virus can cause an independent chain of infection within the non-bovine species. The unintentional exposure of a pregnant goat to a PI heifer and the subsequent birth of a PI kid offered a unique opportunity to expose naive pregnant goats to a PI goat to gain evidence for i) the transmission of BVDV from goat to goat and ii) the generation of PI kids from this transmission. In addition, exposure of pregnant goats to the PI heifer permitted us to investigate the consequences of cattle to goat and the goat to goat transmissions for the viral genome.

As the seroconversions reveal, all animals in group A were infected within the 7 days exposure period (Table 1). However, all animals in this group lost their foetuses. It has been shown before that abortion is the most likely outcome when pregnant goats are exposed to BVDV [30,31]. In case of foetus A3_1, other common causes of abortion in goats were excluded such as Chlamydia and Neospora. In addition, unexposed goats kept in the same premises as the exposed goats, did not abort. However, we cannot rule out that the stress of transport, change of environment and the unfamiliar presence of the heifer may have contributed to the abortions. In most cases, possible signs of abortions such as bloody vaginal secretions were observed between days 104 and 111 of gestation (Table 1). However, death of the conceptus may have occurred earlier, as shown in the case of goat A3 that discharged a small, mummified foetus shortly before the end of gestation. None of the other foetuses were found. Therefore, we conclude that they also died early and may have been lysed, resorbed or excreted, and over-seen. Abortions of small or mummified foetuses in late gestation have also been described by Broaddus et al. [30] after BVDV infection of pregnant goats. In contrast to abortions, reports of viable PI offspring generated by cattle to goat transmissions are rare and even though our initial PI goat was the result of such a transmission, we were unable to repeat this event although the pregnant goats were from the same flock and virus was transmitted from the same calf in the same pen.

Only two of the three goats exposed to the PI goat (group B) seroconverted after the 21 days of exposure (Table 1). The differences in experimental set-up and the small numbers of animal make a statistical comparison of the two groups impossible. However, it may well be that cattle spread pestiviruses more efficiently than small ruminants due to more saliva being spread during feed uptake. In an experimental exposure of naive cattle to two border disease PI sheep for 72 days, only six of the nine calves seroconverted which may point to PI small ruminants being relatively poor pestivirus shedders [45]. Even though only two out of three animals in group B seroconverted, the generation of viable PI offspring was rather efficient as both animals generated BVDV positive kids. When newborn, the PI kids (B2_1 and 2) showed slight tremor and ataxia, symptoms that had not been observed in the initial PI goat. Border disease-like symptoms, difficulties to stand, ataxia and general weakness have been described previously in goat kids after BVDV infection during gestation [28,29,33,46]. However, in most cases, the animals died within 24 h after birth. In our case the animals recovered without medical intervention except for being given an infrared lamp. However, similar to the findings in the Austrian PI goat [35], all our PI goats showed growth retardation. Also, ectoparasites were more of a problem than they are in normal goats. A striking sign was the massive anaemia that affected all PI goats at different ages. Anaemia was probably caused by a reduced erythropoiesis, but the exact pathogenesis and the way it is modulated by BVD virus remains unclear. A severe case of anaemia has previously been described in a BVDV PI cow [47], but seems to have been caused by haemolysis rather than by reduced erythropoiesis. Even though two of the three PI goats (the initial PI goat and B1_2) reached the age of 22 and 15 months, respectively, it should be taken into consideration that they were kept under controlled conditions. It remains questionable if, under field conditions, they would have survived long enough to pass the virus on. Recent reports indicate that cattle-independent BVDV infections can be sustained in white-tailed deer by PI fawns even though these PI animals show reduced performance [11,48]. Furthermore, upon experimental transmissions, virus resulting from deer to deer transmission was infectious for cattle [49]. We have not been able to analyse the BVDV transmission from PI goats back to cattle. However, an outbreak of BVDV in a goat herd in Norway that resulted from a contaminated vaccine led to seroconversions and one abortion in cattle kept in the same barn [29].

In vivo and in vitro experiments have shown that the highly variable viral envelope glycoprotein E2 of BVDV is a major determinant of its species tropism [19,50]. Upon interspecies transmission, nucleotide changes in the E2 coding region of the viral genome may quickly become apparent [19], most likely as a result of selection of pre-existing viral variants rather than true adaptive mutations [51,52]. Indeed, nucleotide ambiguities in the E2 sequence of the initial PI goat may point to changes in the composition of the quasispecies in this animal compared to the sequence obtained from the PI heifer, which might facilitate viral propagation in goats. In support of this interpretation, some of these ambiguities progressed to a complete switch of the amino acid in the consensus sequence in the second goat “passage”, i.e. the next generation of PI kids (Figure 3, Additional file 3). The initial PI goat that originated from cattle to goat transmission may thus represent an intermediate state of viral adaptation to the new host. A comparison of growth curves of virus from the PI heifer and the PI goat on caprine cells showed them to be nearly identical [39]. While not excluding slight differences in viral growth as a contributing factor, adaptation to goats may involve mechanisms unrelated to the level of viral replication in goat cells in vitro. Among others, adaptation might involve interactions with the goats’ immune system. However, only the change at nucleotide position 59 falls into the known antigenic domain of the E2 protein and none of the four changes appears to affect any known epitope [53,54]. Similarly, the “sheep-specific” nucleotide changes at positions 9 and 192 described by Paton et al. [19] upon consecutive cattle and sheep infections were unaffected. However, virus from the initial PI goat passaged in vitro showed amino acid changes at the same positions as those observed after goat to goat infection, which might indicate that they did not occur randomly (Figure 3). In contrast to the virus from the initial PI goat, the virus from the PI heifer did not show any changes in the consensus sequence even after 10 passages in bovine or caprine cells (“in vitro”, Figure 3 and Additional file 3), which indicates that a larger number of replication cycles is required to lead to a change in the consensus sequence of the viral quasispecies of this virus.

In summary, our results indicate that PI offspring are rarely generated in goats after contact with BVDV PI cattle. To our knowledge, this is the first report showing that a PI goat cannot only transmit BVD virus to other goats but that such a transmission may lead to the birth of a second generation of PI goats. The changes in the genome of BVD virus transmitted from a PI calf via transiently infected goats to first and second generation PI kids indicate that adaptation to the non-bovine species is correlated with changes in the quasispecies, leading to step-wise changes in the consensus sequence of the virus.

Thus, BVDV PI goats have the potential to initiate an independent chain of infection in their own species. However, even though second generation PI goats may be produced more easily than first generation PI goats, BVDV transmission from PI cattle to goats leads mainly to abortions and, provided that live PI animals are born, the fitness of these animals may be severely reduced. Hence, although BVDV may be able to infect goats transiently without causing serious disease, it is likely that, compared to infection in cattle, the host-pathogen interaction in goats is not sufficiently refined to enable permanent establishment in this species. It is not surprising, therefore, that BVD virus has not been detected as an “emerging goat pathogen” in serological and virological surveys [55]. Although these arguments do not support the view that goats may become a reservoir host for BVD virus that could jeopardise ongoing BVD eradications, small ruminants could nevertheless acquire an undesired role in such programmes. Goats and, even more importantly, sheep are main hosts for border disease virus and diagnostic tests suitable for mass-testing do not clearly differentiate between different pestiviruses or antibodies to pestiviruses. Hence, after eradication of BVDV in cattle, spill-over of BVDV or BDV from goats and sheep could interfere with the monitoring of freedom from BVDV by serological means. Further knowledge on the role of interspecies transmissions of pestiviruses is therefore important, particularly for BVDV eradication programmes.

## Competing interests

The authors declare that they have no competing interests.

## Authors’ contributions

Experimental design and planning: HRV, CB, MS, EP; animal experiments: HRV; viral molecular analyses: CB, HS; serological analyses: RZ; cell culture work: TM; histopathology and immunohistological analyses: MH; data processing and drafting of manuscript: CB. All authors read and approved the manuscript.

## Supplementary Material

Additional file 1 **Graphical overview of exposure groups and individual animal numbers.** Red = virus positive; blue = virus negative; red crosses = goat kids died shortly before parturition. Click here for file

Additional file 2 **Video sequence of PI goat kids.** The footage shows the BVDV PI goat kids B2_1 and B2_2 at the age of 3 hours and 3 weeks post partum. Click here for file

Additional file 3 **Nucleotide alignment of the BVDV E2 coding region.** The coding region for the envelope glycoprotein E2 was determined and sequences of viruses originating from cattle to goat and goat to goat infections were compared to the virus of the PI heifer. In addition, the E2 coding region of the viruses from the PI heifer and the initial PI goat (termed “PI goat”) that had been passaged in vitro in bovine turbinate (BT) and goat synovial membrane cells (GSM) are included. Dots represent identical nucleotides. Nucleotide ambiguities: R = A or G; M = A or C; W = A or T. Click here for file

## References

[B1] Pletnev A Gould E Heinz FX Meyers G Thiel H-J Bukh J Stiasny K Collett MS Becher P Simmonds P Rice CM Monath TP King AMQ, Adams MJ, Carstens EB, Lefkowitz EJ Flaviviridae Virus Taxonomy 2011 9 Oxford: Academic Press 1003 1020

[B2] Johnson DW Muscoplat CC Immunological abnormalities in calves with chronic bovine viral diarrhea Am J Vet Res 1973 34 1139 1141 4747034

[B3] Tråvén M Alenius S Fossum C Larsson B Primary bovine viral diarrhoea virus infection in calves following direct contact with a persistently viraemic calf Zentralbl Veterinarmed B 1991 38 453 462 1719713 10.1111/j.1439-0450.1991.tb00895.x

[B4] Lindberg ALE Alenius S Principles for eradication of bovine viral diarrhoea virus (BVDV) infections in cattle populations Vet Microbiol 1999 64 197 222 10.1016/S0378-1135(98)00270-3 10028173

[B5] Doyle LG Heuschele WP Bovine viral viarrhea virus-infection in captive exotic ruminants J Am Vet Med Assoc 1983 183 1257 1259 6315662

[B6] Van Campen H Ridpath J Williams E Cavender J Edwards J Smith S Sawyer H Isolation of bovine viral diarrhea virus from a free-ranging mule deer in Wyoming J Wildl Dis 2001 37 306 311 11310881 10.7589/0090-3558-37.2.306

[B7] Becher P Orlich M Shannon AD Horner G König M Thiel HJ Phylogenetic analysis of pestiviruses from domestic and wild ruminants J Gen Virol 1997 78 1357 1366 9191930 10.1099/0022-1317-78-6-1357

[B8] Scherer CF Flores EF Weiblen R Caron L Irigoyen LF Neves JP Maciel MN Experimental infection of pregnant ewes with bovine viral diarrhea virus type-2 (BVDV-2): effects on the pregnancy and fetus Vet Microbiol 2001 79 285 299 10.1016/S0378-1135(00)00357-6 11267789

[B9] Terpstra C Wensvoort G A congenital persistent infection of bovine virus diarrhoea virus in pigs: clinical, virological and immunological observations Vet Q 1997 19 97 101 10.1080/01652176.1997.9694750 9323848

[B10] Carman S Carr N DeLay J Baxi M Deregt D Hazlett M Bovine viral diarrhea virus in alpaca: abortion and persistent infection J Vet Diagn Invest 2005 17 589 593 10.1177/104063870501700613 16475521

[B11] Passler T Ditchkoff SS Givens M Brock KV DeYoung RW Walz PH Transmission of bovine viral diarrhea virus among white-tailed deer (*Odocoileus virginianus*) Vet Res 2010 41 20 10.1051/vetres/2009068 19922743 PMC2797653

[B12] Vilček S Paton DJ Rowe LW Anderson EC Typing of pestiviruses from eland in Zimbabwe J Wildl Dis 2000 36 165 168 10682761 10.7589/0090-3558-36.1.165

[B13] Uttenthal Å Grøndahl C Hoyer MJ Houe H van Maanen C Rasmussen TB Larsen LE Persistent BVDV infection in mousedeer infects calves - do we know the reservoirs for BVDV? Prev Vet Med 2005 72 87 91 10.1016/j.prevetmed.2005.08.006 16213611

[B14] Nelson DD Dark MJ Bradway DS Ridpath JF Call N Haruna J Rurangirwa FR Evermann JF Evidence for persistent Bovine viral diarrhea virus infection in a captive mountain goat (*Oreamnos americanus*) J Vet Diagn Invest 2008 20 752 759 10.1177/104063870802000606 18987224

[B15] Liu H Jiang S Isolation and identification of bovine viral diarrhoea mucosal disease virus from sheep Chin Vet Sci Technol 1987 1 5 7

[B16] Carlsson U Border disease in sheep caused by transmission of virus from cattle persistently infected with bovine virus diarrhoea virus Vet Rec 1991 128 145 147 10.1136/vr.128.7.145 1851350

[B17] Hewicker-Trautwein M Liess B Frey HR Trautwein G Virological and pathological findings in sheep fetuses following experimental infection of pregnant ewes with cytopathogenic-bovine-virus diarrhoea virus Zentralbl Veterinarmed B 1994 41 264 276 7839747 10.1111/j.1439-0450.1994.tb00227.x

[B18] Vilček S Nettleton PF Paton DJ Belák S Molecular characterization of ovine pestiviruses J Gen Virol 1997 78 725 735 9129644 10.1099/0022-1317-78-4-725

[B19] Paton D Gunn M Sands J Yapp F Drew T Vilček S Edwards S Establishment of serial persistent infections with bovine viral diarrhoea virus in cattle and sheep and changes in epitope expression related to host species Arch Virol 1997 142 929 938 10.1007/s007050050129 9191858

[B20] Giangaspero M Harasawa R Genetic variety of bovine viral diarrhea virus 2 strains isolated from sheep J Vet Med Sci 2004 66 323 326 10.1292/jvms.66.323 15107567

[B21] Mishra N Rajukumar K Vilček S Tiwari A Satav JS Dubey SC Molecular characterization of bovine viral diarrhea virus type 2 isolate originating from a native Indian sheep (*Ovies aries*) Vet Microbiol 2008 130 88 98 10.1016/j.vetmic.2008.01.005 18308487

[B22] Mishra N Rajukumar K Tiwari A Nema RK Behera SP Satav JS Dubey SC Prevalence of Bovine viral diarrhoea virus (BVDV) antibodies among sheep and goats in India Trop Anim Health Prod 2009 41 1231 1239 10.1007/s11250-009-9305-z 19153817

[B23] Løken T Pestivirus infections in Norway - epidemiologic studies in goats J Comp Pathol 1990 103 1 10 10.1016/S0021-9975(08)80130-2 2168445

[B24] Krametter-Froetscher R Loitsch A Kohler H Schleiner A Schiefer P Moestl K Golja F Baumgartner W Prevalence of antibodies to pestiviruses in goats in Austria J Vet Med B Infect Dis Vet Public Health 2006 53 48 50 10.1111/j.1439-0450.2006.00906.x 16460357

[B25] Danuser R Vogt HR Kaufmann T Peterhans E Zanoni R Seroprevalence and characterization of pestivirus infections in small ruminants and new world camelids in Switzerland Schweiz Arch Tierheilkd 2009 151 109 117 10.1024/0036-7281.151.3.109 19263380

[B26] Czopowicz M Kaba J Schirrmeier H Bagnicka E Szaluś-Jordanow O Nowicki M Witkowski L Frymus T Serological evidence for BVDV-1 infection in goats in Poland Acta Vet Hung 2011 59 399 404 10.1556/AVet.2011.022 21727071

[B27] Krametter-Froetscher R Loitsch A Moestl K Sommerfeld-Stur I Baumgartner W Seroprevalence of border disease and bovine viral diarrhoea in sheep and goats in selected regions of Austria Wien Tierarztl Monatsschr 2005 92 238 244

[B28] Løken T Bjerkas I Experimental pestivirus infections in pregnant goats J Comp Pathol 1991 105 123 140 10.1016/S0021-9975(08)80068-0 1663955

[B29] Løken T Krogsrud J Bjerkas I Outbreaks of border disease in goats induced by a pestivirus-contaminated Orf vaccine, with virus transmission to sheep and cattle J Comp Pathol 1991 104 195 209 10.1016/S0021-9975(08)80103-X 1650802

[B30] Broaddus C Lamm C Kapil S Dawson L Holyoak G Bovine viral diarrhea virus abortion in goats housed with persistently infected cattle Vet Pathol 2009 46 45 53 10.1354/vp.46-1-45 19112114

[B31] Depner KR Hübschle OJ Liess B Transplacental BVD virus transmission after experimental inoculation of goats in different pregnancy stages Dtsch Tierarztl Wochenschr 1990 97 421 423 (in German) 2245784

[B32] Mishra N Dubey R Rajukumar K Tosh C Tiwari A Pitale SS Pradhan HK Genetic and antigenic characterization of bovine viral diarrhea virus type 2 isolated from Indian goats (*Capra hircus*) Vet Microbiol 2007 124 340 347 10.1016/j.vetmic.2007.04.023 17509780

[B33] Kim IJ Hyun BH Shin JH Lee KK Lee KW Cho KO Kang MI Identification of bovine viral diarrhea virus type 2 in Korean native goat (*Capra hircus*) Virus Res 2006 121 103 106 10.1016/j.virusres.2006.04.008 16766076

[B34] Krametter-Froetscher R Benetka V Duenser M Bagó Z Theiner A Preyler B Moestl K Vilcek S Baumgartner W Descriptive study of a pestivirus infection in an Austrian goat Vet Rec 2008 163 192 194 10.1136/vr.163.6.192 18689783

[B35] Krametter-Froetscher R Schmitz C Benetka V Bago Z Moestl K Vanek E Baumgartner W First descriptive study of an outbreak of Border disease in a sheep flock in Austria - a high risk factor for Bovine viral diarrhea virus free cattle herds: a case report Vet Med (Praha) 2008 53 625 628

[B36] Løken T Bjerkas I Larsen HJ Experimental pestivirus infections in newborn goat kids J Comp Pathol 1990 103 277 288 10.1016/S0021-9975(08)80048-5 2175318

[B37] Løken T Tempesta M Border disease in goats Recent Advances in Goat Diseases 2000 Ithaca NY: International Veterinary Information Service [http://www.ivis.org]

[B38] Hilbe M Stalder H Peterhans E Haessig M Nussbaumer M Egli C Schelp C Zlinszky K Ehrensperger F Comparison of five diagnostic methods for detecting bovine viral diarrhea virus infection in calves J Vet Diagn Invest 2007 19 28 34 10.1177/104063870701900105 17459829

[B39] Mathys T Interspecies transmission of pestivirus: host tropism and adaptation of BVDV and BDV in bovine, caprine and ovine cells as an in vitro model 2007 Switzerland: DVM thesis. University of Bern, Institute of Veterinary Virology

[B40] Bachofen C Stalder H Braun U Hilbe M Ehrensperger F Peterhans E Co-existence of genetically and antigenically diverse bovine viral diarrhoea viruses in an endemic situation Vet Microbiol 2008 131 93 102 10.1016/j.vetmic.2008.02.023 18424020

[B41] Vilček S Development of PCR tests for the detection of bovine herpesvirus-1, bovine respiratory syncytial viruses and pestiviruses Vet Med (Praha) 1994 39 687 700 (in Slovak) 7817501

[B42] Canal CW Strasser M Hertig C Masuda A Peterhans E Detection of antibodies to bovine viral diarrhoea virus (BVDV) and characterization of genomes of BVDV from Brazil Vet Microbiol 1998 63 85 97 10.1016/S0378-1135(98)00232-6 9850989

[B43] Hilbe M Arquint A Schaller P Zlinszky K Braun U Peterhans E Ehrensperger F Immunohistochemical diagnosis of persistent infection with bovine viral diarrhea virus (BVDV) on skin biopsies Schweiz Arch Tierheilkd 2007 149 337 344 10.1024/0036-7281.149.8.337 17803113

[B44] Müller-Doblies D Arquint A Schaller P Heegaard PM Hilbe M Albini S Abril C Tobler K Ehrensperger F Peterhans E Ackermann M Metzler A Innate immune responses of calves during transient infection with a noncytopathic strain of bovine viral diarrhea virus Clin Diagn Lab Immunol 2004 11 302 312 15013980 10.1128/CDLI.11.2.302-312.2004 PMC371222

[B45] Reichle SF Clinical and virological investigations of calves exposed to border disease persistently infected sheep 2009 Switzerland: DVM thesis, University of Zurich, Department for Farm Animals

[B46] Depner K Hübschle OJ Liess B BVD-virus infection in goats - experimental studies on transplacental transmissibility of the virus and its effect on reproduction Arch Virol Suppl 1991 3 253 256 10.1007/978-3-7091-9153-8_31 9210949

[B47] Braun U Jehle W Hilbe M Peterhans E Lutz H Haemolytic anaemia in a heifer with bovine viral diarrhoea and mucosal disease complex Vet Rec 2005 157 452 453 16215249 10.1136/vr.157.15.452

[B48] Negrón ME Pogranichniy RM Van Alstine W Hilton WM Lévy M Raizman EA Evaluation of horizontal transmission of bovine viral diarrhea virus type 1a from experimentally infected white-tailed deer fawns (*Odocoileus virginianus*) to colostrum-deprived calves Am J Vet Res 2012 73 257 262 10.2460/ajvr.73.2.257 22280387

[B49] Raizman EA Pogranichniy RM Lévy M Negrón M Van Alstine W Experimental infection of colostrum-deprived calves with bovine viral diarrhea virus type 1a isolated from free-ranging white-tailed deer (*Odocoileus virginianus*) Can J Vet Res 2011 75 65 68 21461198 PMC3003565

[B50] Liang D Sainz IF Ansari IH Gil LH Vassilev V Donis RO The envelope glycoprotein E2 is a determinant of cell culture tropism in ruminant pestiviruses J Gen Virol 2003 84 1269 1274 10.1099/vir.0.18557-0 12692293

[B51] Jones LR Zandomeni R Weber EL Quasispecies in the 5’ untranslated genomic region of bovine viral diarrhoea virus from a single individual J Gen Virol 2002 83 2161 2168 12185269 10.1099/0022-1317-83-9-2161

[B52] Collins ME Desport M Brownlie J Bovine viral diarrhea virus quasispecies during persistent infection Virology 1999 259 85 98 10.1006/viro.1999.9697 10364492

[B53] Paton DJ Lowings JP Barrett ADT Epitope mapping of the gp53 envelope protein of bovine viral diarrhea virus Virology 1992 190 763 772 10.1016/0042-6822(92)90914-B 1381537

[B54] Deregt D Bolin SR van den Hurk J Ridpath JF Gilbert SA Mapping of a type 1-specific and a type-common epitope on the E2 (gp53) protein of bovine viral diarrhea virus with neutralization escape mutants Virus Res 1998 53 81 90 10.1016/S0168-1702(97)00129-9 9617771

[B55] Menzies PI Control of important causes of infectious abortion in sheep and goats Vet Clin North Am Food Anim Pract 2011 27 81 93 10.1016/j.cvfa.2010.10.011 21215892

