# Supplementary figures and images for: Persistent infections after natural transmission of bovine viral diarrhoea virus from cattle to goats and among goats
Source: Vet Res. 2013 May 15;44(1):32. doi: 10.1186/1297-9716-44-32 (PMC3660168; doi:10.1186/1297-9716-44-32)

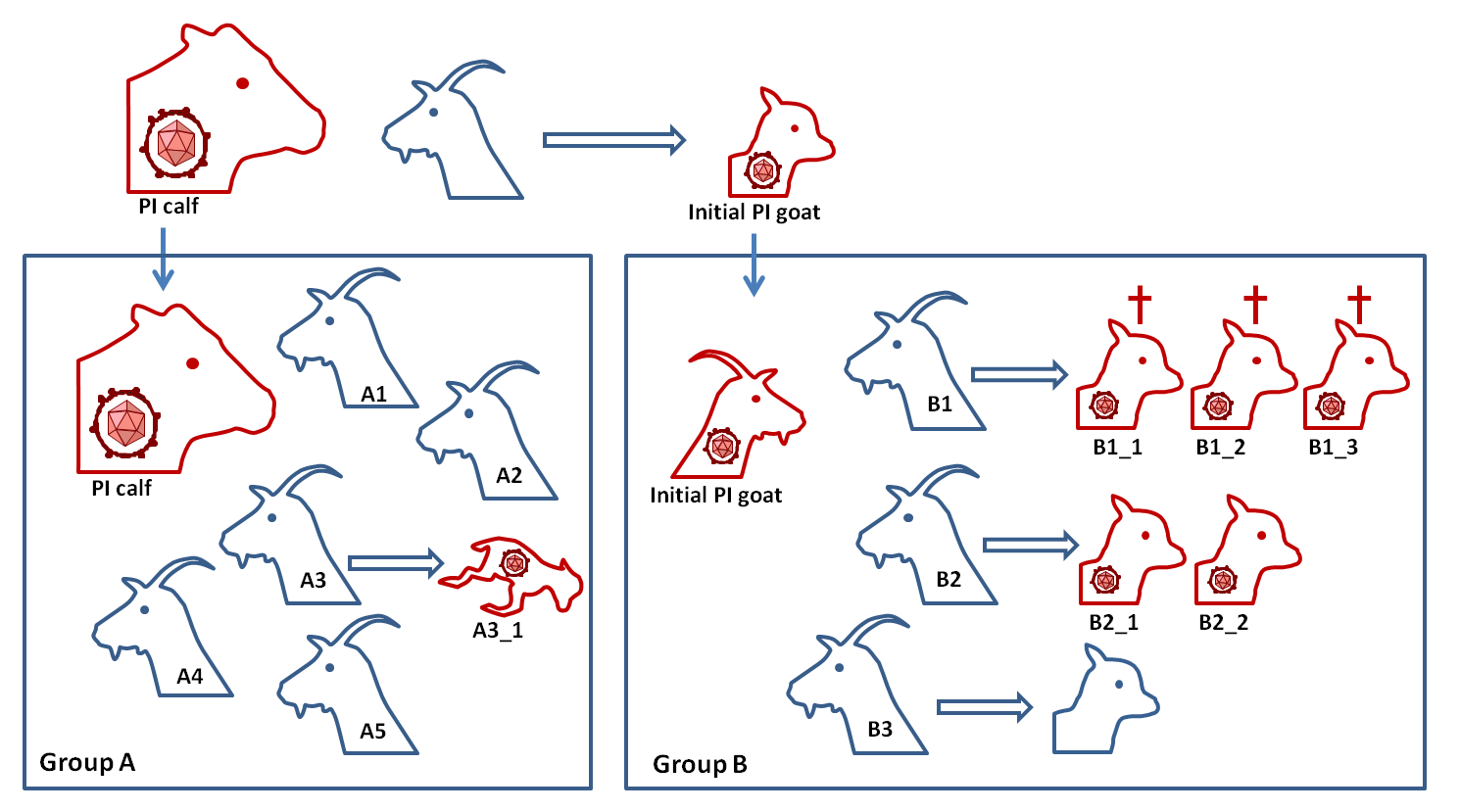

Supplement: Additional file 1 — Graphical overview of exposure groups and individual animal numbers. Red = virus positive; blue = virus negative; red crosses = goat kids died shortly before parturition. [file 1297-9716-44-32-S1.png]

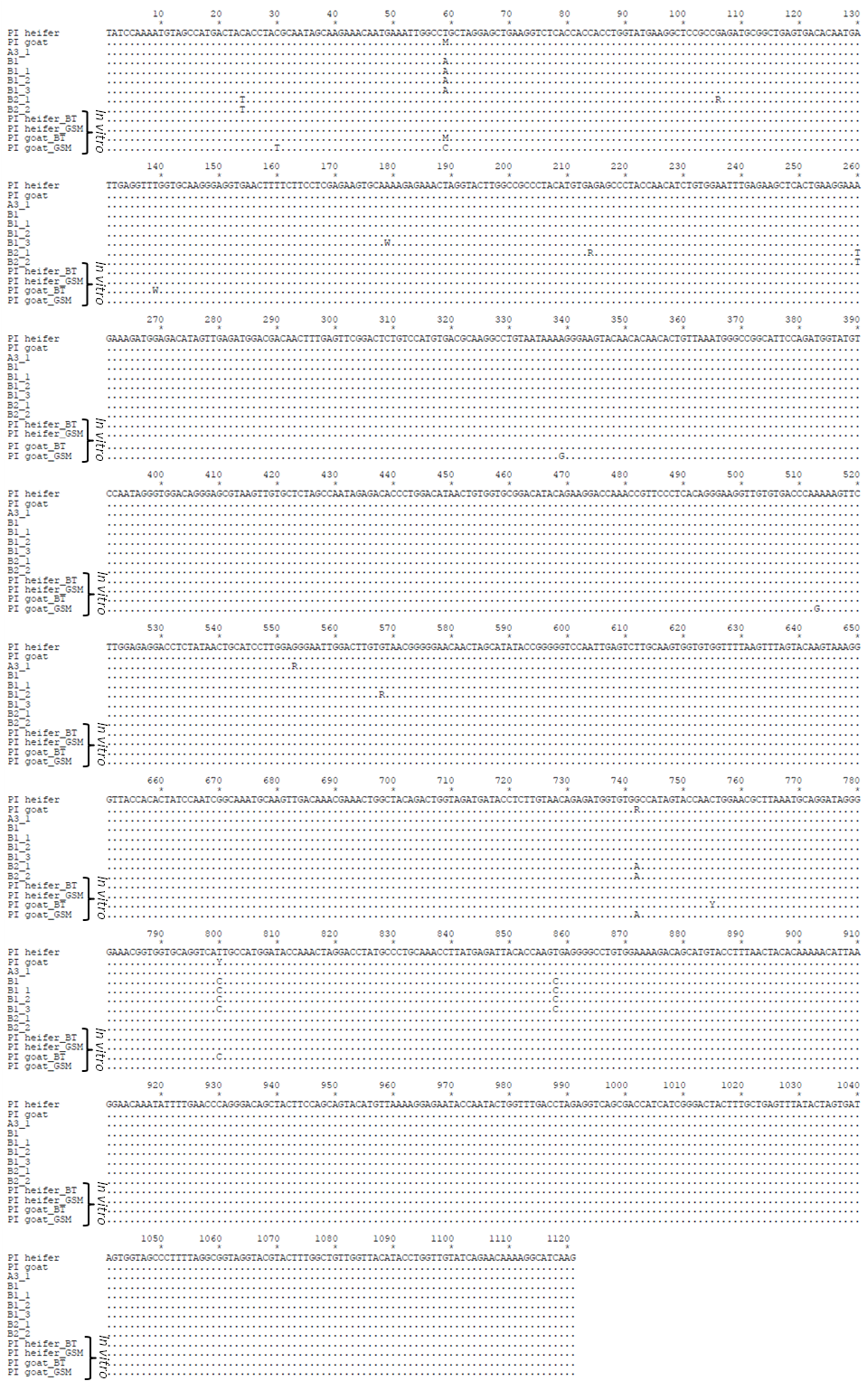

Supplement: Additional file 3 — Nucleotide alignment of the BVDV E2 coding region. The coding region for the envelope glycoprotein E2 was determined and sequences of viruses originating from cattle to goat and goat to goat infections were compared to the virus of the PI heifer. In addition, the E2 coding region of the viruses from the PI heifer and the initial PI goat (termed “PI goat”) that had been passaged in vitro in bovine turbinate (BT) and goat synovial membrane cells (GSM) are included. Dots represent identical nucleotides. Nucleotide ambiguities: R = A or G; M = A or C; W = A or T. [file 1297-9716-44-32-S3.png]
